# Molecular mechanisms of experience‐dependent maturation in cortical GABAergic inhibition

**DOI:** 10.1111/jnc.14103

**Published:** 2017-07-11

**Authors:** M. Ridzwana Begum, Judy C. G. Sng

**Affiliations:** ^1^ Department of Pharmacology Yong Loo Lin School of Medicine National University of Singapore Singapore Singapore

**Keywords:** critical period, epigenetics, inhibitory interneurons, parvalbumin, perineuronal nets, plasticity

## Abstract

Critical periods (CP) in early post‐natal life are periods of plasticity during which the neuronal circuitry is most receptive to environmental stimuli. These early experiences translate to a more permanent and sophisticated neuronal connection in the adult brain systems. Multiple studies have pointed to the development of inhibitory circuitry as one of the central factors for the onset of critical periods. We discuss several molecular mechanisms regulating inhibitory circuit maturation and CP, from gene transcription level to protein signaling level. Also, beyond the level of gene sequences, we briefly consider recent information on dynamic epigenetic regulation of gene expression through histone methylation and acetylation and their implication on timed development of the inhibitory circuitry for the onset of CP.

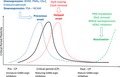

**Abbreviations used**
2‐AG: 2‐arachidonoylglycerol
5HT3aR: serotonin receptor 5HT3a
BDNF: brain‐derived neurotrophic factor
CNS: central nervous system
CP: critical period
CPSGs: chondroitin sulfate proteoglycans
DAG: diacylglycerol
DGL: DAG‐lipase
ECM: extracellular matrix
GABA: gamma‐aminobutyric acid
HAT: histone acetyltransferase
HDAC: histone deacetylases
iLTD: long‐term depression of inhibitory transmission
MD: monocular deprivation
NCAM: neural cell adhesion molecule
Otx2: orthodentricle homeobox 2
PNN: perineuronal nets
PRMT: protein arginine methyltransferases
proBDNF: precursor of BDNF
PSA: polysialic acid
PST: poly‐siayltransferases
PV+: parvalbumin‐expressing, fast‐spiking GABAergic
PV: parvalbumin
sIPSC: spontaneous inhibitory postsynaptic currents
SST: somatostatin
STD: short‐term depression
TnR: tenascin R

Neuronal circuits are exceptionally sensitive to being shaped by patterns of cellular activity during early brain development called critical periods (Hensch 2005a). The critical period (CP) refers to a defined developmental window when the system is especially sensitive to environmental stimuli and experience produces permanent, large‐scale changes to neural circuits. CPs are known to be present in sensory systems such as binocular vision in the visual cortex, barrel representation of whiskers in somatosensory cortex, and tonotopic map refinement in auditory cortex. They are also present in motor systems and even areas of higher cognitions in the brain such as human language acquisition in the Broca's area (O'Leary *et al*. 1994; Hensch 2004, 2005a; Knudsen 2004; Daw 2006; Hooks and Chen 2007).

The aim of studies investigating mechanisms underlying activation and regulation of CPs in the central nervous system (CNS) are to identify mechanisms that may allow for reactivation of neural circuit plasticity in adulthood when these circuits are no longer plastic. An important application would be to aid rewiring of neural circuits after damage to the CNS such as in the case of stroke to regain normal function. In children with neurodevelopmental disorders involving synaptic plasticity, understanding mechanisms to reactivate critical period can also potentially help to correct alterations in neural circuits via pharmacological intervention.

The visual cortex is the best‐studied experimental model used to study critical period as it is easy to manipulate visual experience independently in each eye and measure changes in plasticity. Direct electrophysiological measurements of neuronal function measure ocular dominance, a phenomenon whereby visual cortical neurons are activated to different degrees by the presentation of visual stimuli to one eye versus the other. During the CP, cortical responses can be manipulated by deprivation of sensory inputs via the closure of one eye, known as monocular deprivation (MD), leading to irreversible synaptic reorganization of neural circuits. This in turn results in the decrease in ability of the deprived eye to elicit cortical neuronal responses and in the increase in number of neurons responsive to the open eye. This phenomenon is known as the ocular dominance plasticity, first described in kittens by Nobel award winners Hubel and Wiesel (Hubel and Wiesel 1959, 1962). Aside from MD, there exists CPs and plasticity for orientation and direction selectivity (Wang *et al*. 2010). The closure for the CP of direction selectivity occurs prior to ocular dominance plasticity; however, it is unclear if vision plays an essential role to maintain the orientation selectivity (Butz *et al*. 2009). Pharmacological studies have shown that manipulating the inhibitory–excitatory circuitry can either modify or eliminate directional and orientation selectivity in primary visual cortices of cats (Sillito 1979; Sillito *et al*. 1980; Eysel and Shevelev 1994; Monier *et al*. 2003). The visual cortical circuits exhibit maximal plasticity in young animals during CP and this is lost after this unique period as prolonged visual deprivation in adults does not cause changes in visual cortical responses (Hensch and Fagiolini 2004). Furthermore, the young animals that underwent MD lost visual acuity in the deprived eye permanently, despite exposure of the closed eye to subsequent visual experience (Berardi *et al*. 2000).

Different brain regions, such as the somatosensory cortex and the auditory cortex, have also been used to study other forms of experience‐dependent plasticity exhibiting a critical period. In the somatosensory cortex, CPs for somatosensory mapping occur at different time points for different layers. Moreover, plasticity can be defined as structural plasticity and functional plasticity but is often used interchangeably in papers as they are more than often coupled. Structural plasticity refers to anatomical changes in connectivity between neurons, that is, synaptic rewiring, whereby new synaptic connections are formed and/or number of neurons or synapses are altered. However, functional plasticity refers to the strength of a single synapse that is either strengthened by long‐term potentiation or weakened by long‐term depression thus affecting the function of the existing neurons (Butz *et al*. 2009). In some conditions, they may have varying CPs but the question remains as to what common factors determine the timing of CP across different brain regions.

One of these factors implicated in the onset of CP plasticity appears to be the development of inhibitory circuits (Fagiolini and Hensch 2000; Hensch and Fagiolini 2004; Huang *et al*. 2007). Inhibitory interneurons make up ~ 20% of cortical neurons and they secrete the neurotransmitter gamma‐aminobutyric acid (GABA) (Markram *et al*. 2004). They serve to regulate neuronal excitability (Swadlow 2003), integration (Pouille and Scanziani 2001), generate temporal synchrony, and oscillation among a network of excitatory neurons (Somogyi and Klausberger 2005). Even during development, these interneurons regulate cell migration via GABA (Luhmann *et al*. 2015), differentiation of neurons, and experience‐dependent refinement of neuronal connections (Ben‐Ari 2002a; Hensch and Fagiolini 2004). Although the GABAergic inhibitory interneurons are made of multiple varied populations, three primary populations: the Ca^2^^+^ ‐binding protein parvalbumin (PV), neuropeptide somatostatin, and the ionotropic serotonin receptor 5HT3a (5HT3aR)‐expressing interneurons have been shown to account for almost 100% of GABAergic interneurons in the neocortex (Rudy *et al*. 2011). PV‐expressing interneurons make up 40% of the inhibitory interneuron population, with somatostatin and 5HT3aR‐expressing interneurons each making up 30% of the population (Rudy *et al*. 2011). Several studies have pointed to PV‐expressing interneurons as the ones involved in critical period plasticity regulation**.** The expression of PV in interneurons and critical period onset coincide (Del Rio *et al*. 1994); furthermore, both are accelerated by BDNF over‐expression (Huang *et al*. 1999). Deletion of a potassium current (Kv3.1) that specifically regulates the fast‐spiking behavior of PV‐expressing interneurons slows the rate of ocular dominance plasticity (Hensch 2005b).

The focus of this paper is on the molecular mechanisms regulating post‐natal GABAergic circuit development and CP, all the way from protein signaling in the synapse, gene transcription, and epigenetic regulation of gene expression in the nucleus. Expression of genes such as *Bdnf*,* Otx* (Sugiyama *et al*. 2008), and *Npas4* (Bloodgood *et al*. 2013) are crucial for regulating critical period plasticity by regulating and maintaining maturation of inhibitory circuits and the timed expression of these genes are in turn regulated by epigenetic marks rearranging chromosomal DNA for transcription.

## Molecular mechanisms regulating GABAergic development

### Signaling molecules

#### BDNF

Brain‐derived neurotrophic factor (BDNF) is a growth factor required for the formation of GABAergic synapses in hippocampal and cortical cultures (Rutherford *et al*. 1997; Vicario‐Abejon *et al*. 1998). To investigate if BDNF also has the same function *in vivo,* over‐expression of BDNF in the visual cortex resulted in accelerated development of GABAergic circuits and inhibition which is correlated with a premature onset and closure of the ocular dominance plasticity (Huang *et al*. 1999)^,^(Hanover *et al*. 1999). Furthermore, dark‐reared mice treated with BDNF injections to the retina showed normal expression of GABA and GAD65 unlike untreated dark‐reared mice that exhibited reduced GABA and GAD65 expression (Lee *et al*. 2006).

Interestingly, brain slices treated with mature BDNF protein, but not precursor of BDNF (proBDNF) exhibited decreased inhibition (Frerking *et al*. 1998; Holm *et al*. 2009). As the Bdnf gene can be transcribed via multiple promoters (I–VIII) to produce transcripts with unique 5′ exon (exons I–VIII) that are spliced on to the common 3′ coding exon (exon IX), 9 types of *Bdnf mRNA* transcripts are found in rodents (Aid *et al*. 2007) and 17 in humans (Pruunsild *et al*. 2007). As unique *Bdnf* mRNA transcripts are expressed during developmental time points and are regulated by different factors, it appears that some transcripts are expressed at basal levels required for neuronal survival and differentiation, while others exhibit experience‐dependent *Bdnf* expression responsible for experience‐dependent circuit maturation and plasticity (Hong *et al*. 2008; Sakata *et al*. 2009). To investigate the role of activity‐dependent *Bdnf* expression mediated at promoter IV specifically, Hong *et al*. generated a mouse line that carried a mutation of the CaRE3/CRE(cAMP/Ca^++^‐response element‐like element) in the endogenous promoter IV blocking the its activity‐dependent expression. These mice have decreased spontaneous inhibitory postsynaptic currents (sIPSCs) in cortical culture and lesser GABAergic synapses in the cortex (Hong *et al*. 2008). Another study by Sakata *et al*. disrupted the promoter IV mediated *Bdnf* transcription by inserting a green flourescent protein‐stop cassette after exon IV. The resulting mice had fewer PV‐expressing, fast‐spiking GABAergic interneurons in the prefrontal cortex, reduced frequency and amplitude of sIPSCs in cortical culture (Sakata *et al*. 2009). However, the disruption of experience‐dependent *Bdnf* transcription from promoter IV in both studies did not affect the structure and function of cortical glutamatergic synapses. These studies show that activity‐dependent *Bdnf* transcription is crucial for the development of inhibitory circuits in the cortex.

In order to understand how experience‐dependent *Bdnf* expression regulates development of inhibitory circuits, immunohistochemical studies suggest that BDNF produced in cortical neurons act as an intercellular signaling molecule. BDNF communicates pyramidal neuron activity to GABAergic interneurons which express TrkB receptors specific for BDNF (Cellerino *et al*. 1996; Singh *et al*. 1997; Holm *et al*. 2009). To study the effect of BDNF signaling on the GABAergic circuitry, mutant mice that have a specific deletion of TrkB receptor in parvalbumin‐expressing, fast‐spiking GABAergic (PV+) interneurons were used. These mutant mice exhibited decreased amplitude of glutamatergic inputs to PV+ interneurons and frequency of PV+ interneuron inputs to excitatory pyramidal neurons were also reduced and decreased rhythmic network activity in the gamma frequency band (Zheng *et al*. 2011). Furthermore, treatment with TrkB inhibitor K252‐a showed that the regulation of GABAergic activity was mediated by BDNF signaling via the TrkB receptor (Holm *et al*. 2009).

#### GABA

Unsurprisingly, another signaling molecule that up‐regulates GABAergic synapse maturation is GABA. During early development, GABA is excitatory because of high concentration of intracellular chloride ions. It later transits to become inhibitory via the delayed expression of a specific K^+^‐Cl^−^‐coupled co‐transporter whose expression leads to a negative shift in the reversal potential for chloride ions (Ben‐Ari 2002b). As the GABAergic and glutamatergic synapses are formed sequentially, inhibition eventually becomes necessary for normal function. During this time, intracellular chloride ions are expelled in an activity‐dependent manner and GABA starts to function as an inhibitory neurotransmitter rather than being excitatory (Ben‐Ari *et al*. 1989; Wang and Kriegstein 2009).

GABA is synthesized by two forms of glutamic acid deoxycarboxylase – GAD65 and GAD67, the deletion of either gene reduces GABA levels (Asada *et al*. 1997; Hensch *et al*. 1998). GAD67 is primarily expressed early in development, in the cell body and nerve terminals contributing to ~ 90% of GABA synthesis, whereas the remaining ~10% is synthesized by GAD65 that is expressed later in development and localized to pre‐synaptic terminals (Pinal and Tobin 1998). Previous studies have shown that knockdown of GAD67 in mice resulted in aberrant perisomatic synapse maturation (Chattopadhyaya *et al*. 2007), whereas knockdown of GAD65 results in deficiency for maintaining stable perisomatic synapses (Hensch *et al*. 1998). These results lead the authors to conclude that GABA is required for inhibitory synapse formation. However, in a more recent study, Wu *et al*. demonstrated that knocking out genes encoding for GAD67, GAD65 and vesicular GABA transporter specifically in single basket interneurons, lead to increased bouton and axon density with normal synapse structures when compared to the wildtype interneurons (Wu *et al*. 2012). This study suggests that instead of forming inhibitory synapses, GABA acts to eliminate subsets of synapses in an activity‐dependent manner, while promoting the maturation of other synapses. As GAD67, GAD65 expression is activity‐dependent (Patz *et al*. 2003), GABA functions as an experience‐dependent regulator of inhibitory synapses.

Not only is GABA required for the development of GABAergic synapses, the receptors controlling GABA signaling are also important for synapse development and interneuron axon arborization. GABA signaling is largely mediated by ionotropic GABA_a_ receptors, and to a smaller extent by metabotropic GABA_b_ receptors activated by endogenous release of GABA (McLean *et al*. 1996). Recent studies have shown that GABA_a_ receptors display an excitatory signaling mechanism in Down syndrome and its reversal restores synaptic plasticity in mice (Deidda *et al*. 2015). Also, treatment with GABA_a_ or GABA_b_ receptor agonists, resulted in recovery of perisomatic synapses in GAD67^−/−^ mice (Chattopadhyaya *et al*. 2007). These results suggest the significance of GABA receptors in synapse development. As these receptors are present on post‐synaptic neurons, GABA axon terminals and surrounding glial processes, cell‐autonomous activation of pre‐synaptic GABA_b_ receptors (modulating Ca^2+^ channels and GABA release) influences growth cone motility and bouton stability. Furthermore, GABA signaling through post‐synaptic or glia receptors could trigger retrograde factors, promoting axon branching and synapse formation. Fiorentino *et al*., demonstrated that one of the possible mechanisms utilized by GABA to regulate synapse formation is via the metabotropic GABA_b_ receptors on pyramidal neurons that trigger secretion of BDNF and promotes the development of perisomatic GABAergic synapses in hippocampal neurons (Fiorentino *et al*. 2009). In addition, GABA_a_ receptors appear to be negatively regulated by the precursor of BDNF (proBDNF) – p75 signaling pathway. The presence of proBDNF results in degradation of GABA_a_ receptors and repression of its synthesis resulting in decreased inhibitory transmission. The cleavage of proBDNF to mature BDNF is mediated by tissue‐type plasminogen activator and the expression of tissue‐type plasminogen activator is found to be implicated in experience‐dependent plasticity in the visual system (Mataga *et al*. 2004). Insofar, these studies suggest that there is a positive interplay between experience‐dependent activity, BDNF, and GABA signaling for the maturation of GABAergic synapses.

Aside from receptors, GABA transporters regulate signaling between synapses. GABA released by pre‐synaptic terminals is not enzymatically broken down and instead, its clearance depends completely on diffusion and its uptake by specific transporters, which therefore regulates the activation of GABA receptors (Scimemi 2014). In mice, there exist four pharmacologically distinct GABA transporters (GAT1 – GAT4), of which GAT1 and GAT4 are brain specific. Early studies show that GAT1 and GAT4 expression correlates with the α1 subunit of GABA receptors (Jursky and Nelson 1996), which is found to drive cortical plasticity (Fagiolini *et al*. 2004). In rats, GAT 1 and GAT3 display post‐natal changes that reflect the maturation of GABAergic inhibition (Vitellaro‐Zuccarello *et al*. 2003). These observations together suggest that the transporters may have an implication of the maturation of GABAergic innervation and synaptic plasticity; however, much research has not been conducted to further elucidate its association.

### Endocannabinoid signaling

Endocannabinoids are produced and released post‐synaptically and act as retrograde negative regulators of pre‐synaptic neurotransmitter release (Chevaleyre *et al*. 2006; Lovinger 2008; Kano *et al*. 2009). They bind to cannabinoid receptors (CB1R) pre‐synaptically and mediate long‐term depression of inhibitory transmission (iLTD) underlying the decrease in release probability at inhibitory synapses of fast‐spiking PV+ interneurons during development in the visual cortex (Jiang *et al*. 2010a). iLTD was found to be induced only during experience‐dependent critical period of the layer II/III visual cortex. When antagonists against CB1R were applied or CB1RKO mice were used, absence of endocannabinoid‐induced iLTD prevented the characteristic decrease in release probability, short‐term depression, and response variability in mature cortical GABAergic inhibition (Jiang *et al*. 2010a).

In a follow up study, the authors found that there exists a laminar difference in sensitivity to endocannabinoids in the visual cortex (Jiang *et al*. 2010b; Sun *et al*. 2015). They showed that GABAergic synapses in layer II/III and layer V were more sensitive to the CB1R agonist showing precocious maturation and this was not observed in transgenic CB1R knockout mice, demonstrating that endocannabinoid signaling is responsible for iLTD. However, in layer IV, administration of CB1R agonist at any age did not result in precocious maturation. This suggests that although endocannabinoids do not play a role in maturation of GABAergic inhibition in layer IV of the visual cortex, they may be partly responsible for maturation of GABAergic inhibition in layer II/III and V. These findings also provide an explanation for the differences in timing in maturation of GABAergic inhibitory circuits between layer IV and layers II/III and V (Jiang *et al*. 2010b; Sun *et al*. 2015).

**Figure 1:**
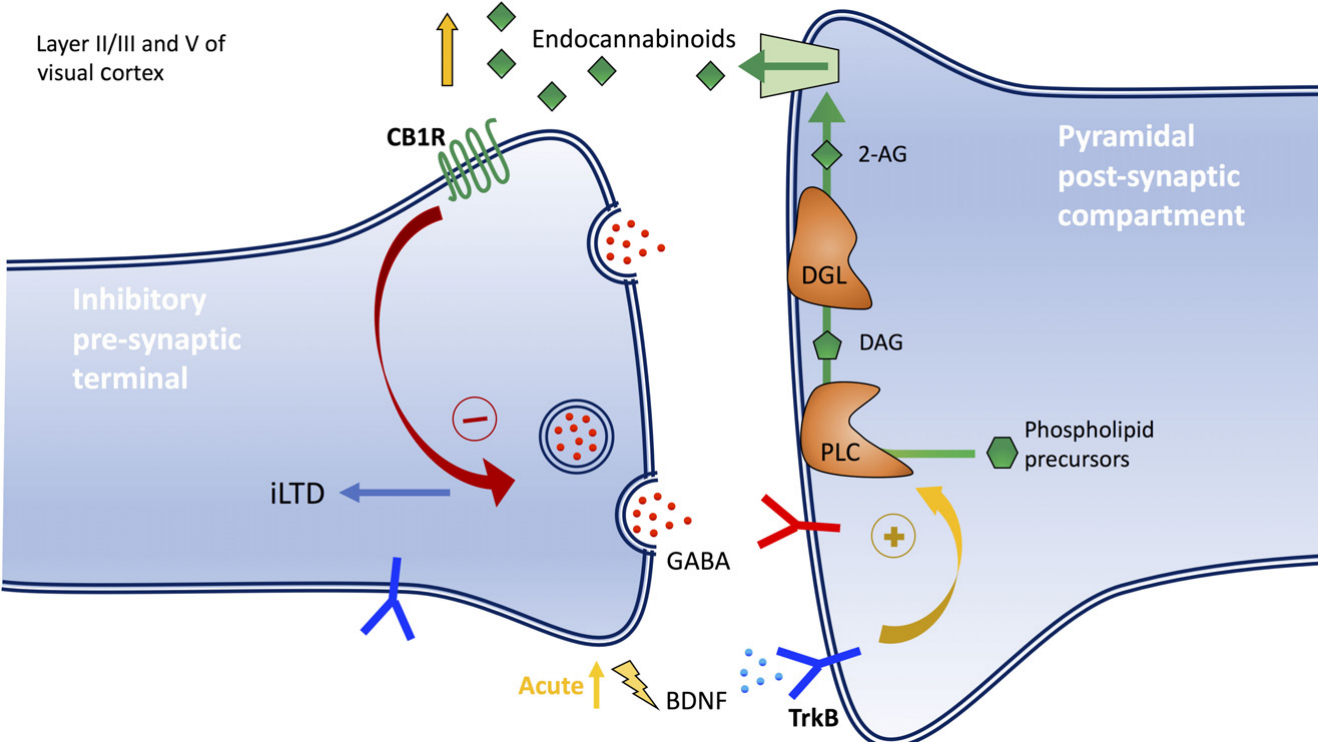
Endocannabinoid‐mediated long‐term depression of inhibitory transmission (iLTD) in GABAergic pre‐synaptic terminal induced by endogenous brain‐derived neurotrophic factor (BDNF)‐TrkB signaling during the critical period. Retrograde signaling of endocannabinoids from post‐synaptic pyramidal compartments negatively regulates GABA release from the pre‐synaptic membrane and subsequently causes maturation of the inhibitory circuitry through long‐term depression of inhibitory transmission (iLTD). An acute increase in endogenous BDNF decreases GABA release and promotes iLTD. Phospholipase C (PLC)‐diacylglycerol lipase (DGL) pathway generates the CB1R agonist 2‐arachidonoylglycerol (2‐AG). First, the precursor of 2‐AG, diacylglycerol (DAG) is generated by membrane‐associated PLC that cleaves the phosphate group from the membrane phospholipid precursors when in contact. DAG is then hydrolyzed by DGL to form 2‐AG which is released from the post‐synaptic compartment for retrograde signaling in the pre‐synaptic terminal. The exact mechanism for its release is still unclear. The endocannabinoids then bind to CB1R and sends feedback to decrease the secretion of GABA neurotransmitters**.**

Recent studies have shown that BDNF can induce endocannabinoid release and there exist cross‐talk between BDNF and endocannabinoid signaling. Evidence of interaction between endocannabinoids and BDNF interaction are found in the visual cortex (Huang *et al*. 2008), hippocampus (Khaspekov *et al*. 2004), and cerebellum (Maison *et al*. 2009). In addition, studies have also shown that TrkB receptors and CB1R are strongly colocalized throughout the forebrain, layer II/III and V of the cortex (Cabelli *et al*. 1996; Fryer *et al*. 1996; Miller and Pitts 2000), with the highest levels of CB1R found in layer II/III (Matsuda *et al*. 1993; Tsou *et al*. 1998; Marsicano and Lutz 1999; Egertová *et al*. 2003). Endocannabinoid synthesis and release was found to be mobilized by BDNF‐TrkB signaling, acute application of BDNF in the cortex suppressed pre‐synaptic GABA release, causing decreased GABAergic transmission. This decrease in GABA release was because of retrograde signaling of endocannabinoids from the post‐synaptic pyramidal neuron (Fig. 1) (Lemtiri‐Chlieh and Levine 2010). The production of endocannabinoid synthesis is independent of mGluR and is initiated by post‐synaptic TrkB signaling followed by downstream Phospholipase C (PLC) signaling (Zhao and Levine 2014). Endogenous BDNF‐TrkB signaling is required for inducing endocannabinoid‐mediated iLTD that occurs during the critical period for the maturation of GABAergic inhibition. Blockade of both the TrkB receptors and the activation of diacylglycerol lipase (DAG‐lipase, DGL) abolished iLTD at layer II/III cortical inhibitory synapses suggesting that the endocannabinoid 2‐arachidonoylglycerol (2‐AG) is involved in the signaling pathway (Zhao *et al*. 2015). However, sufficient evidence is not available to completely rule out the involvement of other endocannabinoids such as anandamide.

### The extracellular matrix (ECM): holding it all together

The extracellular matrix makes up a huge portion of the brain volume (Ruoslahti 1996). The extracellular matrix (ECM) not only provides physical support for the cells in the nervous system, they are also hypothesized to play crucial roles in neurotransmission of signals. A remarkable feature of the brain is its capacity to remodel itself to changing neuronal activity. As a result, remodeling or pruning occurs at the level of synapses. A major role of these ECM is to modulate plasticity by rapid reorganization of synaptic connections during the critical period of development. Such is the importance of the ECMs that artificial removal by pharmacological intervention leads to reactivation of neural plasticity. Here, we describe two groups of molecules that interact with the extracellular environment to regulate synaptic remodeling.

#### Polysialic acid (PSA) – NCAM

Neural cell adhesion molecule (NCAM) is a key player in cell–cell adhesion of neuronal circuits (Rutishauser 2008). In the mammalian brain, NCAM is a unique substrate for poly‐siayltransferases (PST). PST attaches polymers of **α**‐2,8‐linked sialic acid onto NCAMs, causing the NCAMs to lose its adhesive properties (Cunningham *et al*. 1983; Sadoul *et al*. 1983). Addition of polysialic acid (PSA) onto NCAMs has been implicated in many neuronal processes, such as migration (Ono *et al*. 1994), axon guidance (Tang *et al*. 1992; Seki and Rutishauser 1998; El Maarouf and Rutishauser 2003), and synaptogenesis (Dityatev *et al*. 2004). It is hypothesized that PSA acts as a switch, functionally switching NCAM between a cell adhesion molecule and a signaling molecule. In early development, PSA binds to NCAMs, permitting cell migration to happen. This is followed by the development of neurite processes and even synaptogenesis. Once these developmental processes are completed, there is an accompanying reduction in expression of PSA. As a signaling molecule, NCAMs are known to interact with a host of signaling molecules, which include Fibroblast growth factor (FGF) receptors (Doherty and Walsh 1996; Rønn *et al*. 1999), Glial cell line‐derived neurotrophic factor (GDNF) (Paratcha *et al*. 2003), and neurotrophin receptors such as BDNF (Muller *et al*. 2000).

Mouse models of NCAMs or PSTs deletion have been shown to cause deficits in synaptic plasticity and cognitive function. This makes PSA‐NCAMs an attractive candidate in the study of synaptic plasticity. In a study published by Di Cristo *et*. *al*., the authors discovered that PSA is down‐regulated after visual experience but dark‐rearing attenuates this down‐regulation. Moreover, enzymatic removal of PSA causes early onset of critical period in the visual cortex by enhancing inhibitory synaptic transmission. This inverse relationship of PSA expression and timing of maturation of GABAergic innervation (Di Cristo *et al*. 2007) makes PSA a key player in functional and structural development of GABAergic inhibitory circuits.

#### Perineuronal nets (PNN)

As described previously, inhibitory circuit maturation is pivotal for critical period plasticity. Studies have shown, in both visual and somatosensory modalities, that a special kind of ECM known as perineuronal nets (PNN) form around inhibitory neurons, specifically PV+ neurons (Brückner *et al*. 1993; Härtig *et al*. 1994). Perineuronal nets are extracellular structures made of many glycoprotein components, namely chondroitin sulfate proteoglycans, hyaluron ,and tenascins (TnR and TnC) (Köppe *et al*. 1997; Carulli *et al*. 2006; Deepa *et al*. 2006). PNNs form around the soma and proximal dendrites of PV interneurons and they influence the synapse development. This is functionally important as the PNNs consolidate the connections established during development and prevents any changes to the synapses. Studies have shown that components of the PNNs have activity‐dependent expression (Sur *et al*. 1988; Lander *et al*. 1997; Kind *et al*. 2013; Ye and Miao 2013), suggesting the important role that they play in maintaining synapses and neurotransmission. Interestingly, the expression of PNNs is concomitant to a decrease in plasticity, suggesting that PNNs consolidate the matured state of synaptic connections (Hensch 2003) by acting as a structural brake. Not surprisingly, if one were to reduce or remove activity by deprivation paradigms, such as dark‐rearing (Hockfield *et al*. 1990) or monocular deprivation (Sur *et al*. 1988; Pizzorusso *et al*. 2002, 2006) and whisker trimming (McRae *et al*. 2007; Nakamura *et al*. 2009; Nowicka *et al*. 2009), PNN expression will be reduced and plasticity is maintained. This is supported by evidence that removal of PNNs recovers ocular dominance plasticity in the visual system (Pizzorusso *et al*. 2002) of adult rats.

Besides acting as a structural brake, PNNs also have a role in providing a microenvironment for molecular cues such as Semaphorin 3A (Vo *et al*. 2013) and orthodenticle homeobox 2 (Otx2) (Sugiyama *et al*. 2008; Beurdeley *et al*. 2012). Otx2 will be discussed further in the section below.

Taken together, the ECM provides permissive cues for plasticity in the brain by physical methods and also provides the platform for instructive cues such as transcription factors to act on the cell.

### Transcription factors

#### Orthodenticle homeobox 2 (Otx2)

Otx2 is a transcription factor whose expression is selectively in the retina and then when triggered by visual experience is transferred from cell to cell until it reaches the PV interneurons and is accumulated (Sugiyama *et al*. 2008). When the visual cortex was infused with Otx2, PV interneuron maturation and critical period closure were both accelerated. However, conditional removal of Otx2 from the visual pathway resulted in the loss of critical period plasticity (Sugiyama *et al*. 2008). This suggests that Otx2 is also required for opening the critical period.

**Figure 2:**
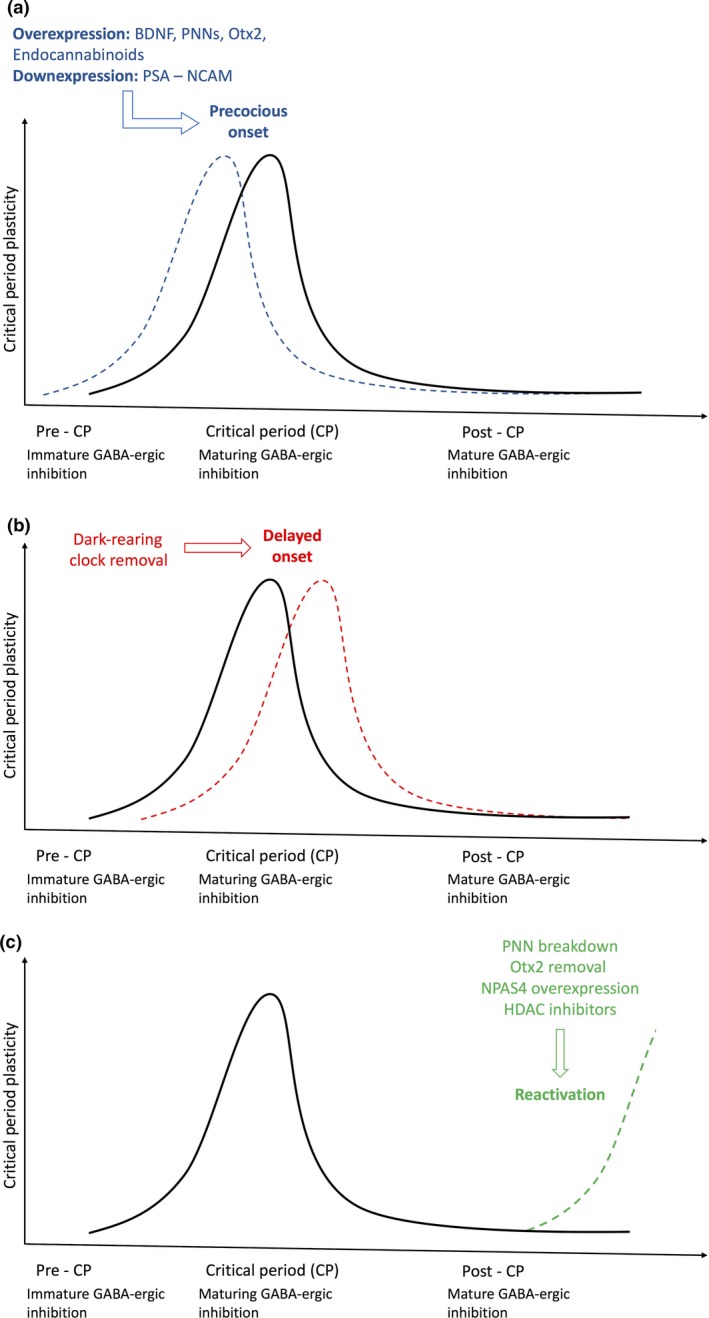
Factors affecting the onset and reactivation of experience‐dependent critical period plasticity. (a) Early manipulations in pre‐critical period can allow for the precocious onset of plasticity primarily by hastening the maturation of the GABAergic circuitry. Blue dotted lines indicate the shift in the onset, peak, and closure of critical period to an earlier timing. An increase in either brain‐derived neurotrophic factor (BDNF), endocannabinoids, perineuronal nets (PNN) formation, or orthodenticle homeobox 2 (Otx2), or a decrease in polysialic acid (PSA)‐neural cell adhesion molecule (NCAM) interaction leads to an increase in inhibition in neuronal circuitry which in turn has shown to trigger the early onset of critical period plasticity. (b) Behavioral and genetic interventions such as dark‐rearing (specifically for visual plasticity) and Clock removal during pre‐critical period have been implicated in delaying the onset of critical period to a later age as shown by the red dotted lines. (c) Interventions for reactivation of plasticity in adulthood post‐critical period during which neuronal circuitry has already been consolidated. Counteracting particular brakes on plasticity through pharmacological approaches (HDAC inhibitors, chondroitinase ABC for PNN breakdown) or genetic approaches (Otx removal, NPAS4 over‐expression) has shown to reactivate plasticity as represented by the green dotted lines.

Otx2 bind to the aforementioned PNNs wrapped around the surface of the PV interneurons. These PNNs constitutively capture Otx2, enabling the accumulation of Otx2 in PV interneurons. The action of PNNs was demonstrated by hydrolysis of PNNs by chondroitinase ABC and via blockade of the binding motif of Otx2 to PNNs. Preventing Otx2 from binding to PNNs resulted in the reactivation of critical period plasticity in adult mice (Fig. 2). Hence, constant accumulation of Otx2 is required to maintain the closure of the critical period (Beurdeley *et al*. 2012).

#### NPAS4

NPAS4 is a transcription factor whose expression is activated by excitatory synaptic activity. Subsequently, it triggers downstream gene expression for the formation and maintenance of inhibitory synapses on excitatory neurons and regulates experience‐dependent GABAergic synapse development (Lin *et al*. 2008). The expression of NPAS4 parallels that of visual cortical plasticity and over‐expression of NPAS4 reactivated critical period plasticity in adult mice (Fig. 2). However, when NPAS4 was knocked down in combination with fluoxetine, the reactivation of plasticity in adulthood initially caused by fluoxetine, was prevented. The results suggest that NPAS4 regulates downstream expression of synaptic plasticity genes essential for reactivation (Maya‐Vetencourt *et al*. 2012).

#### Clock

Clock is a transcription factor known to regulate circadian rhythms that regulate many physiological processes (Lowrey and Takahashi 2011). A recent study has shown that it is also implicated in timing the maturation of PV interneurons (Kobayashi *et al*. 2015). In Clock knockout mice, not only was circadian gene expression decreased but the maturation of PV interneurons was also delayed. These transgenic mice additionally exhibited late onset and extended critical period plasticity. To study if Clock directly regulates PV interneurons, specific deletion of Clock in PV neurons only displayed the same outcome as Clock knockout mice resulting in the late onset of critical period (Fig. 2) (Kobayashi *et al*. 2015). Collectively, these results show that Clock impacts PV interneurons directly and affect their maturation.

### Epigenetics: more than ATGC

There is a dynamic interplay between genes and experience, nature versus nurture, a clearly delineated and biochemically driven mechanistic interface known as epigenetics. Epigenetics allows post‐mitotic, non‐dividing neurons to dynamically regulate chromatin state. The state of chromatin packing determines how accessible gene regulatory elements are and how they can be regulated in response to environmental cues. Besides rapid and dynamic control over gene regulation, it is becoming clearer that epigenetics allows multiple permutations of ‘genetic codes’ without drastically increasing the actual amount of genomic material in each cell. This is supported by the fact that the human genome is no larger than the genome of lesser evolved organisms (Lander *et al*. 2001; Venter *et al*. 2001). This ‘epigenome’ alters regulation of genetic material, is reversible, and does not alter the primary DNA sequence. Some examples of common epigenetic marks are DNA cytosine methylation, histone acetylation/deacetylation, protein methylation, and phosphorylation.

In fact, the ENCODE project (Bernstein *et al*. 2012; Consortium RE, Kundaje A, Meuleman W, *et al*., 2015) was launched to study the epigenomes of human brain tissues and to uncover the role of ‘neuroepigenetics’ in regulation of neuronal function. As such it is not surprising that the maturation of GABAergic interneurons are dependent on timed expression of various genes. Epigenetic regulators such as DNA methylation, hydroxymethylation, post‐translational histone modifications, histone variants, and spatial arrangement of highly condensed DNA play an important role in regulating downstream signaling for the development of GABAergic circuits during the critical period and its maintenance. Here, we describe two interesting and emerging types of epigenetic marks that studies have shown may play important roles in PV+ inhibitory interneuron function.

#### HDAC inhibitors

Many studies on epigenetic mechanisms regulating critical period plasticity are done in the visual cortex. Perhaps, the most well‐studied epigenetic mark for critical period regulation is histone acetylation. Histone acetylation levels are kept in equilibrium by histone acetyltransferases and deacetylases (HDACs) that have opposing actions. An experience‐dependent epigenetic mark, acetylated histone, is abundant at loose chromatin sites of active gene transcription and these marks also exhibit a correlative decrease as critical period plasticity ends (Putignano *et al*. 2007). Administration of HDAC inhibitors reactivates critical period plasticity in the visual cortex (Putignano *et al*. 2007; Vetencourt *et al*. 2011) and also recovers binocular vision in amblyopic adult mice (Silingardi *et al*. 2010). Furthermore, the reactivation of critical period plasticity was also accompanied by a decrease in GABAergic transmission and also increased histone acetylation (H3K9) at *Bdnf* promoter I (Vetencourt *et al*. 2011). The reactivation of critical period plasticity in these studies was correlated with increased histone acetylation after treatment with HDAC inhibitors. In another study, it was also found that there is a relationship between extinction of conditioned fear, histone modification by HDAC inhibitors, specifically valporic acid, and regulation of BDNF gene expression (Bredy *et al*. 2007). Because of the unspecific effects of HDAC inhibitors, it is unclear if the reactivation of plasticity and long‐term extinction of fear is a result of increased histone acetylation or as a result of other molecular mechanisms. To investigate if increase in histone acetylation levels is directly responsible for the reactivation of critical period plasticity, transgenic mice with specific HDAC knockout can be used.

#### HDAC1

**Figure 3:**
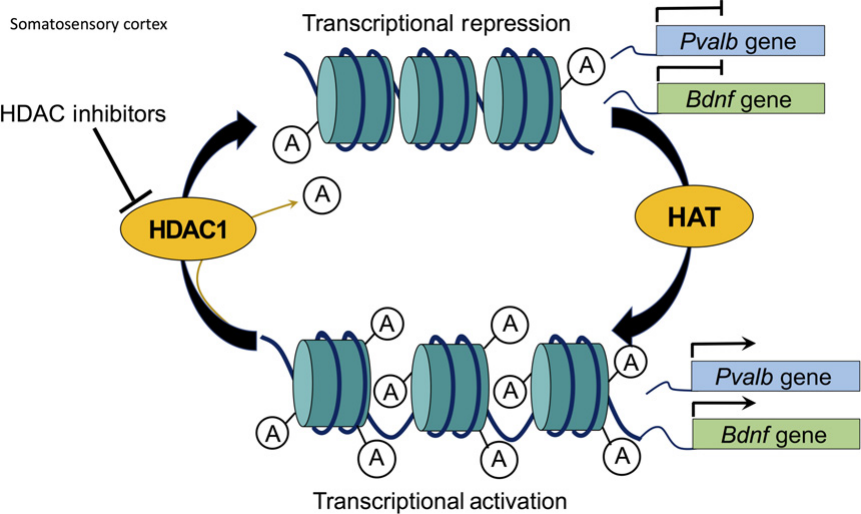
Effect of HDAC1 activity on parvalbumin (pvalb) and brain‐derived growth factor (bdnf) gene expressions in the somatosensory cortex of mice. Histone deacetylation by HDAC1 in promoter regions of Pvalb and Bdnf genes during whisker deprivation results in the transcriptional repression of the genes. Parvalbumin (PV) and brain‐derived neurotrophic factor (BDNF) proteins are implicated in the maturation of inhibitory circuitry, thus affecting the critical period plasticity. Temporal knockdown of HDAC1 specifically recovers Pvalb and Bdnf gene expression through transcriptional activation during whisker deprivation.

The effect of HDAC1 on inhibitory circuitry maturation was quite recently explored in the somatosensory cortex of mice. A decrease in experience‐dependent activation of S1 revealed a negative regulation of *Bdnf* and Parvalbumin (*Pvalb*) genes by HDAC1. Whisker‐deprived animals showed an increase in HDAC1 expression and activity with a corresponding decrease in inhibitory synapses of PV interneurons. Chromatin Immunoprecipitation analysis revealed direct associations between HDAC1 and promoter regions of *Bdnf* and *Pvalb* which were down‐regulated because of the increased histone deacetylation (Fig. 3). A temporal knockdown of HDAC1 recovers *Bdnf* and *Pvalb* expression levels in whisker‐deprived animals and also prevented the decrease in inhibitory synapses. These results collectively demonstrate that HDAC1 plays a role in the development of PV interneurons both through *Bdnf* expression as well as epigenetic regulation of specific genes (Koh and Sng 2016)**.**

#### HDAC2

HDAC2 has previously been shown to regulate synaptic plasticity and formation of memories in hippocampal excitatory neurons; HDAC2 knockout mice have increased synaptic connections and facilitated memory formation (Guan *et al*. 2009). To find out if the maturation of PV+ interneurons (necessary for the closure of critical period plasticity) is dependent on epigenetic regulation, Nott *et al*. deleted HDAC2 specifically in PV+ interneurons (Nott *et al*. 2015). They found that inhibition was reduced and iLTD that typically occurs during the critical period was present in adult mice. These results suggest that HDAC2 is required for the maturation of PV inhibitory interneurons and the closure of the critical period (Nott *et al*. 2015).

#### Protein methylation

Protein methylation, specifically, protein arginine methylation has been implicated in transcriptional regulation, mRNA processing, nuclear‐cytoplasmic shuttling, DNA repair, and signal transduction. An important family of enzymes, the protein arginine methyltranferases (PRMT), has been associated with many cellular processes, mainly in cell cycle progression. Prmts can catalyze the formation of mono‐ or di‐methylated arginine residues. Depending on the type of arginine dimethylation, PRMTs can be classified as a Type I or Type II enzyme (Bedford and Clarke 2009; Wolf 2009). As a consequence of modification to histones, which alters biochemical properties of nucleosomes, chromatin structure and gene regulation is altered.

PRMTs are strongly implicated in stem cell and cancer biology. Also, many groups are beginning to look to PRMTs as potential therapeutic targets (Cha and Jho 2012), such is the importance of PRMTs as a regulatory element. However, the role of PRMTs has not been elicited in neuronal processes such as synaptic plasticity. Of particular interest might be PRMT8, which is specifically located within the central nervous system (Taneda *et al*. 2007; Kousaka *et al*. 2009).

### PRMT8S

**Figure 4:**
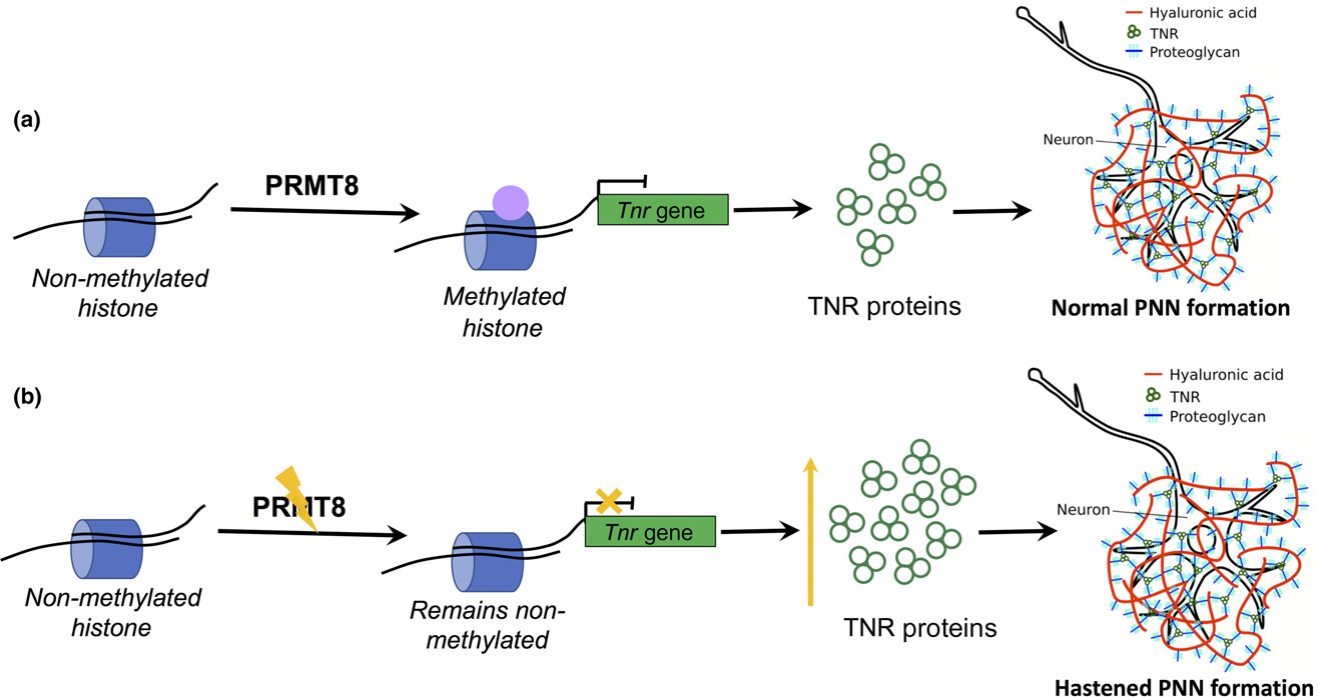
Methylation of arginine residues on histones by protein arginine methyltransferases (PRMT)8. (a) Methylation by PRMTs can either lead to transcriptional activation or transcriptional repression. In the case of the structural protein Tenascin‐R (TNR), methylation by PRMT8 suppresses its expression. TNR proteins are crucial in extracellular complexes organization for the formation of perineuronal nets (PNN). PNNs wrap around parvalbumin (PV) interneurons and consolidate neuronal circuitry acting as a structural break for critical period plasticity. (b) PRMT knockouts lack this suppression of the *Tnr* gene resulting in the increase in TNR protein synthesis. This in effect hastens PNN formation in mice visual cortex resorting to the premature closure of critical period.

Because of its unique tissue localization, PRMT8 is suspected to play an important role in neurodevelopment and synaptogenesis. There are evidence to suggest that it plays a role in early neuronal development (Lin *et al*. 2013). A recent study has shown that PRMT8 is an epigenetic regulator of specific structural proteins, such as Tenascin‐R, that are involved in the formation of perineuronal nets (PNNs). Development of PNNs, as mentioned earlier, closes the critical period plasticity via consolidation of the inhibitory neuronal connections. In *Prmt8* knockout mice, *Tnr* transcription levels were heightened by 1.5‐fold resulting in the increased PNN formation (Fig. 4). Also, there was a 10% increase in wrapping of PV interneurons in PNNs, thus affecting dendritic morphology of the interneurons in knockouts compared to wildtype. Lee *et al*. moreover, showed that the visual acuity of *Prmt8* knockout mice were lower than wildtype mice suggesting a premature closure of critical period because of the hastened PNNs formation. These findings suggest that PRMT8 methylation activity is critical for the complete maturation of inhibitory circuitry by regulating the formation of PNNs that essentially act as structural brakes to critical period plasticity (Lee *et al*. 2017).

## Concluding remarks

With more models to study critical period plasticity emerging in different modalities, GABAergic PV expression has been shown to be a key regulator of critical period plasticity. However, experiences can regulate the maturation of PV+ interneurons via different pathways and studying these processes in different modalities may help us to better understand the differences in regulation between modalities. Further work has to be done to find out if similar pathways of regulation are common in different regions of the brain. Neuroepigenetics is an emerging field crucial for the downstream regulation of gene expression required for PV+ interneuron maturation and can perhaps be used as targets of pharmaceutical intervention as lines of evidence show that using HDAC inhibitors have reactivated critical period plasticity in the visual cortex (Putignano *et al*. 2007; Vetencourt *et al*. 2011) and improved absolute pitch perception in human subjects (Gervain *et al*. 2013). In all, increasing our understanding of such molecular mechanisms regulating developmental plasticity in the brain will enable us to design better pharmaceuticals with lesser side effects aimed to reactivate neural wiring after injury and develop targeted pharmaceuticals to correct alterations in the brain of children suffering from neurodevelopmental disorders caused by imbalance of excitatory‐inhibitory transmission.
